# A narrative approach to trichotillomania

**DOI:** 10.1192/j.eurpsy.2022.1647

**Published:** 2022-09-01

**Authors:** J. Gonçalves Cerejeira, I. Santos Carrasco, A. Gonzaga Ramírez, M. Queipo De Llano De La Viuda, G. Guerra Valera, C. De Andrés Lobo, T. Jiménez Aparicio

**Affiliations:** 1 Hospital Clínico Universitario de Valladolid, Psychiatry, Valladolid, Spain; 2 Clinical Hospital of Valladolid, Psychiatry, Valladolid, Spain

**Keywords:** Trichotillomania, OCD, Narrative Therapy

## Abstract

**Introduction:**

Trichotillomania is an obsessive-compulsive spectrum disorder characterized by recurrent and uncontrolled hair pulling. This behavior causes significant anxiety as well as low self-esteem in people who suffer from this disorder. There is still no therapy of proven efficacy in the treatment of trichotillomania. Psychotropic drugs and cognitive behavioral psychotherapy have been tried in the management of this disease, but the relapse rate is high. Narrative therapy is an innovative type of postmodern psychotherapy and in our literary search we have not found data related to its use in the treatment of trichotillomania.

**Objectives:**

To present a novel therapeutic approach to a highly resistant disorder, trichotillomania.

**Methods:**

Case report and literature review.

**Results:**

We present a case of a 39-year-old woman diagnosed with trichoticolomania twenty years earlier. She tried several types of psychotherapies for manage her hair-pulling problem, all related with relapse only a few days after finishing the sessions. We have carried out a total of 5 narrative therapy sessions spread over 3 months. No relapses have been observed during the subsequent 9-month follow-up period.

**Conclusions:**

Based on our experience, we believe that Narrative Therapy is a good and still unexplored alternative for people diagnosed with trichotillomania.

**Disclosure:**

No significant relationships.

